# Rapid prioritisation of topics for rapid evaluation: the case of innovations in adult social care and social work

**DOI:** 10.1186/s12961-021-00693-2

**Published:** 2021-03-10

**Authors:** Katherine Cowan, Naomi J. Fulop, Amelia Harshfield, Pei Li Ng, Antiopi Ntouva, Manbinder Sidhu, Jon Sussex, Sonila M. Tomini, Holly Walton

**Affiliations:** 1 Katherine Cowan Consulting Limited, St Leonards, UK; 2 Department of Applied Health Research, University College London, Gower Street, London, WC1E 6BT UK; 3 RAND Europe, Westbrook Centre, Milton Road, Cambridge, CB4 1YG UK; 4 Public Health England, 5 St Philip’s Place, Birmingham, B3 2PW UK; 5 Health Services Management Centre, University of Birmingham, Park House, 40 Edgbaston Road, Birmingham, B15 2TT UK; 6 NIHR BioResource, Department of Public Health and Primary Care, University of Cambridge, Cambridge Blood Donor Centre, Long Road, Cambridge, CB2 0PT UK

**Keywords:** Social care, Prioritisation, Rapid, Adults, Innovations

## Abstract

**Background:**

Prioritisation processes are widely used in healthcare research and increasingly in social care research. Previous research has recommended using consensus development methods for inclusive research agenda setting. This research has highlighted the need for transparent and systematic methods for priority setting. Yet there has been little research on how to conduct prioritisation processes using rapid methods. This is a particular concern when prioritisation needs to happen rapidly. This paper aims to describe and discuss a process of rapidly identifying and prioritising a shortlist of innovations for rapid evaluation applied in the field of adult social care and social work.

**Method:**

We adapted the James Lind Alliance approach to priority setting for rapid use. We followed four stages: (1) Identified a long list of innovations, (2) Developed shortlisting criteria, (3) Grouped and sifted innovations, and (4) Prioritised innovations in a multi-stakeholder workshop (*n* = 23). Project initiation through to completion of the final report took four months.

**Results:**

Twenty innovations were included in the final shortlist (out of 158 suggested innovations). The top five innovations for evaluation were identified and findings highlighted key themes which influenced prioritisation. The top five priorities (listed here in alphabetical order) were: Care coordination for dementia in the community, family group conferencing, Greenwich prisons social care, local area coordination and MySense.Ai. Feedback from workshop participants (*n* = 15) highlighted tensions from using a rapid process (e.g. challenges of reaching consensus in one workshop).

**Conclusion:**

The method outlined in this manuscript can be used to rapidly prioritise innovations for evaluation in a feasible and robust way. We outline some implications and compromises of rapid prioritisation processes for future users of this approach to consider.

**Supplementary Information:**

The online version contains supplementary material available at 10.1186/s12961-021-00693-2.

## Background and objectives

This paper describes and discusses a process developed for rapidly identifying and prioritising a shortlist of innovations for rapid evaluation, while engaging with a full range of stakeholder perspectives including service users, carers, practitioners, and service commissioners and providers, and relevant national organisations. Rapid prioritisation is relevant wherever a balance of speed and rigour is sought to enable rapid evidence creation to guide resourcing decisions [1]. We applied this process to adult social care and social work innovations. In this manuscript, we refer to innovations that target adult social care broadly, e.g. new models of care, service innovations, payment and commissioning innovations, person and community-centred approaches to innovations and technological innovations. This approach was undertaken in respect of England but is relevant anywhere.

The majority of innovations that have been piloted and implemented in adult social care and social work in England are mostly small in scale and/or inconsistently implemented [2]. It is essential to learn more about their effectiveness, cost-effectiveness, context and generalisability when considering their potential to be rolled-out more widely [3]. High quality and timely evaluation is needed to identify which innovations are priorities for adoption and scale-up [3]. Given the large number of innovations that could be evaluated, it is necessary to prioritise where to focus limited resources that are available to undertake evaluations.

Identification and prioritisation processes are more widely conducted in healthcare research and evaluation than in social care. It is important to note that there are likely differences between priority setting for research compared to priority setting for evaluation and implementation (e.g. different priority setting exercises may require different criteria and nuanced applications). There is a substantial, international literature on priority setting in healthcare research, but there is currently no agreed gold standard approach [4–7]. Yoshida et al. highlighted the need for a “transparent, replicable, systematic and structured approach” to priority setting [6]. Viergever et al. identified nine best practice themes when conducting health priority setting exercises: context, use of a comprehensive approach, inclusiveness, information gathering, planning for implementation, selection of relevant criteria, methods for deciding on priorities, evaluation and transparency [5]. The WHO reviewed its health research priorities using a research cycle framework [7]. Recognising the need for transparent reporting of priority setting, Tong et al. developed the REporting guideline for PRIority SEtting of health research (REPRISE) guideline, which covers 10 domains: context and scope, governance and team, framework for priority setting, stakeholders/participants, identification and collection of priorities, prioritization of research topics, output, evaluation and feedback, translation and implementation, and funding and conflict of interest [8].

Prioritisation of research and evaluation in social care and social work has attracted less attention than in healthcare, but that may be changing. Increasing demand and constraints in public spending have put social care and social work services in England under severe pressure and that is heightening the need for service evaluations. The Health Foundation and The King’s Fund estimates that demand for publicly funded social care will increase in real terms by 3.7% annually on average over the period to 2030–2031, which is much faster than historic growth in public funding [9]. In response, local government, care providers and other organisations have been looking at novel approaches to deliver services. The Local Government Association’s Green Paper for adult social care and wellbeing [10] and other reports such as “Six Innovations in Social Care” [11] and “Total Transformation of care and support” [12] highlight innovations across the UK in: demand management; working in closer partnership with the National Health Service (NHS), voluntary and social enterprise sectors; and using community-based assets to provide care solutions to local populations [10, 12]. Government statements on the future of social care in England highlight the importance of innovation [13], and in 2019 the Department of Health and Social Care (England) funded the development of the Social Care Innovation Network, a collaboration between the Social Care Institute for Excellence (SCIE), Think Local Act Personal (TLAP) and Shared Lives Plus to support local providers, commissioners and citizens to adopt evidence-based innovations in social care [14, 15].

Whilst priority setting exercises are more numerous in healthcare research, there are also examples in social care and social work. The James Lind Alliance (JLA) Adult Social Work Priority Setting Partnership (PSP) identified in 2018 the top 10 priority research questions in adult social work in England using the JLA’s long established PSP approach for the first time in a non-health related setting [16]. The approach included stakeholders from the adult social care workforce and providers, but also service users and their carers [16]. In 2019, the National Institute for Health Research (NIHR) undertook a scoping review of adult social care research priorities to guide decisions about funding further research in the area. Thirty distinct research priorities were identified from National Institute for Health and Care Excellence guidelines, NIHR-funded reviews and research, JLA PSPs and other documents [17]. Stakeholder engagement is necessary when conducting priority setting processes as there can be differences between researcher priorities for research topics and end user priorities for research topics [18]. Additionally, stakeholder engagement may increase relevance and reduce research waste [19].

To supplement the JLA and NIHR exercises, which focused on research questions, and to identify priorities specifically for immediate rapid evaluation in adult social care and social work, in July 2019 the NIHR commissioned the rapid prioritisation exercise presented in this paper.

Our rapid prioritisation process aimed to identify and prioritise adult social care and social work innovations for evaluation. This manuscript describes and explains the rapid prioritisation method that we used.

## Methods

Our approach to rapid prioritisation of social care innovations (for subsequent rapid evaluation) focused on achieving speed while retaining an acceptable level of coverage (of the range of social care and social work innovations) and reliability (participation by all key stakeholder groups in the prioritisation process). To achieve this balance, we adapted the JLA approach to priority setting, which uses a dialogue model for multi-stakeholder involvement [18, 20]. The JLA model draws on and adapts consensus development models such as the Nominal group Technique and Delphi methods [21, 22]. Our adapted approach followed four steps: (1) identification of innovations; (2) development of shortlisting criteria; (3) grouping and sifting innovations; and (4) prioritisation of innovations in a multi-stakeholder workshop (See Additional file 1: S1). The whole process (including project initiation through to completion of the final report) took four months (July–November 2019).

We started by identifying a long list of specific, named innovations, rather than topics/questions for research, as would be more conventional in a JLA context. We identified specific innovations rather than underlying topics/questions for research as the purpose of this prioritisation process was to rapidly identify specific innovations for evaluation in the short term. The horizon scanning encompassed all types of innovation in adult social care and social work, including: new models of care; service innovations; payment and commissioning innovations; person- and community-centred approaches; and technological innovations. Emails were sent to 182 individuals or organisations with knowledge of social care and social work, including people who use adult social care services, carers, frontline professionals, service providers, commissioners, national organisations, think tanks and researchers. The stakeholder list was created using the combined networks of all the authors and colleagues (including their research units, contacts of contacts, Google searches and forwarding of the email to interested parties). The email asked stakeholders to identify interesting innovations in adult social care and/or social work, which would benefit from being evaluated. Stakeholders were asked to provide, within a four-week deadline (compared to three months or more in the usual JLA process): the innovation(s)’s name(s); short description; where/who is implementing the innovation; brief description of any known evaluations of the innovation; and links to further information. As part of this exercise, we were recommended to include the innovations in the ‘Six Innovations in Social Care’ report by Think Local Act Personal [11]. Two team members (HW/SMT) grouped innovations which were identical or similar, based on information from the recommender(s) and/or further information from rapid web searches by team members (PLN/HW/SMT). Innovations were grouped into nine categories: (1) Workforce capacity building innovations, (2) Training and support innovations, (3) Technology innovations to support care, (4) Housing community innovations, (5) Home adaptation innovations, (6) Relationship based innovations, (7) Innovations linking patients with health or social care professionals for provision of care, (8) Innovations on social services in the community and (9) Innovations relating to the provision of funding support.

To shorten the (very) long list of innovations to a reduced list, we developed shortlisting criteria. To be included, innovations had to fit within our scope, focus on adult social care and social work, take place within the four nations of the UK, provide enough detail to understand what the innovation is, focus on social care and social work, be amenable to evaluation and rapid evaluation and focus on a relevant outcome for social care (see Additional file 1: S2). To develop criteria, we drew on literature on research prioritisation in health and social care and their own experiences of prioritisation. Criteria were discussed and refined in follow-up team discussions (see Additional file 1: S2).

The criteria were initially applied to the resultant list of innovations by two team members (HW/SMT) in consultation with an expert social care academic adviser independent of the team (CN). We were as inclusive as possible at this stage. Innovations were automatically omitted only where all three of the initial reviewers advised that. To further condense the remaining list of innovations, the full project team met in September 2019. The discussion focused on innovations where there had been disagreement among the three initial reviewers, but also reconsidered those innovations where all three had so far agreed to retain them. Innovations were excluded by the full team at this stage if, when considered by the larger group, it was known that: they had already been evaluated thoroughly; they were mainly healthcare rather than social care/social work focussed; they were not innovative; or were too non-specific. A final reduced list of 20 adult social care and social work innovations was then taken to a multi-stakeholder workshop to identify the top five priorities for evaluation.

A key element of the rapid prioritisation was to engage in depth with a full range of stakeholder perspectives in an open and purposeful discussion to arrive at a well-founded shortlist of adult social care and social work innovations for evaluation. To achieve this, we ran a one-day multi-stakeholder workshop in October 2019, in London. We aimed to recruit around 25 workshop participants including: people who use adult social care services, carers, practitioners, providers, commissioners, researchers and key national organisations. To identify participants, we included an invitation to the workshop in the initial request for innovations (described earlier) and encouraged recipients to pass on the invitation to other individuals likely to be interested. Twenty-three people (beyond the project team) took part in the workshop. Travel costs were covered for participants. People who use adult social care services and carers were offered payment for preparation, workshop attendance and travel time. It was agreed that participants would not be identified in any reporting.

The workshop materials—agenda, participant worksheets and workshop guide—were prepared by an expert practitioner in using the JLA approach, who also led the facilitation of the workshop itself (KC). The format of the workshop was adapted from the JLA model [23]. Prior to the workshop, participants were sent an approximately 200-word description, plus a web link for further information where available, for each of the 20 innovations. Participants were asked to read these and rank all 20 from most to least important to evaluate. These initial views were to be shared with other participants at the start of the workshop.

At the start of the workshop, participants received short presentations which explained its purpose and how the 20 innovations had been identified. Participants were given the opportunity to ask questions and seek clarification. During the workshop, participants were at different times split into three sub-groups with similar numbers of members and a balanced range of stakeholder perspectives. Three facilitators (KC, NJF and JS) guided participants through group activities in which participants discussed and prioritised the list of 20 innovations for evaluations. Facilitators were neutral and did not contribute to discussions or prioritisations.

The work on the day was undertaken in three successive stages of prioritisation, each building on the one before. The criteria for prioritising one innovation ahead of another for evaluation were deliberately not pre-set but were left to the workshop participants to propose (explicitly or implicitly). An initial discussion took place within each small group. Participants took turns to describe their top three and bottom three priorities for evaluation from the list of 20 and their reasoning. The facilitator of each group then summarised and presented back to the group the aggregate of their initial proposals for the highest and lowest priorities to evaluate. The first round of prioritisation then took place, within the same small groups. Drawing on the prior discussion, the facilitator in each group arranged 20 cards (with the individual innovations outlined) to create a diamond shape. The top of the diamond represented the most important innovations expressed in the previous discussion and the lower tip the less important topics. The middle reflected innovations that received divided opinions. The diamond was then developed into a more linear and prioritised list through discussion and negotiation, with all innovations ranked one to 20 by each small group separately. The three groups’ rankings were then combined in a spreadsheet and presented back to all the workshop participants in a plenary session. One facilitator (KC) gave an overview of the combination of all small group rankings, drawing attention to areas of agreement or disagreement between the groups.

In the next prioritisation session, participants were allocated to three new small groups. Thus, participants were with a largely different group from that with which they had discussed priorities in the preceding session. This provided an opportunity for participants to hear and understand different views, and to review and, if agreed, revise the shared ranked list. Participants were advised to focus on the top half of the combined list from the plenary session, in order to work towards a final prioritisation. Again, the three groups’ rankings were entered on a spreadsheet.

In the final prioritisation session, all workshop participants met in plenary to review the aggregate of the second round of group rankings and completed a final round of prioritisation together. This focused on agreeing the top five priorities. It was agreed that the ranking positions of the remaining 15 innovations were of less importance.

Three observers (JE, HW and JN) took notes throughout the workshop. Notes covered how decisions were made, areas of agreement and disagreement, key themes and insights into participants’ perceptions of innovations and their importance for evaluation. These notes were used to provide further insight into why decisions were made and why certain innovations were prioritised over others.

To better understand the participants’ perspectives on the prioritisation workshop process, we asked them to complete feedback forms. Feedback forms included questions on: the stakeholders role at the workshop, how they found the information sent in advance of the workshop, the extent to which the prioritisation process was helpful in agreeing priorities, whether they felt able to voice their opinions, whether everyone was encouraged to join in equally, fairness and independence of facilitators, suitability of the venue and refreshments. Stakeholders were also given the opportunity to provide free-text comments.

## Results

**Fig. 1:**
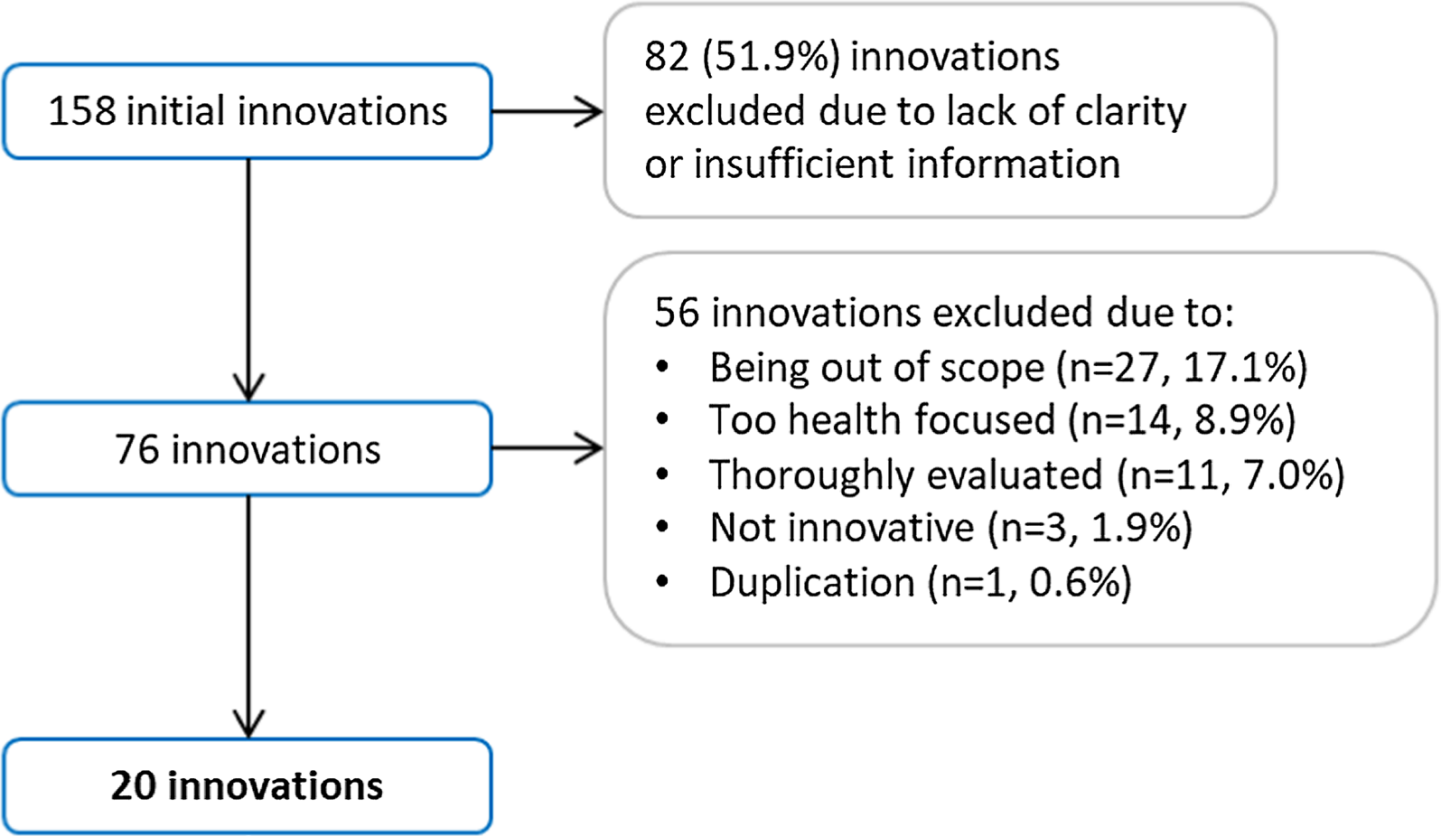
Flow chart demonstrating the inclusion/exclusion of innovations

In total, 158 different innovations were suggested by 59 individuals from 43 academic, government, NHS and third sector organisations. After grouping and sifting by the project team, 20 innovations (12.7% of the original total) were included in the final shortlist (see Fig. 1).

Table 1 indicates the stakeholder groups from which the 23 workshop participants came. There was strong representation by service users and care practitioners, in particular.

**Table 1:** Types and numbers of participants at the prioritisation workshop

Type of participant	Number of attendees
People who use adult social care services	7
Practitioner	6
Academic/researcher	4
Commissioner or policy maker	3
Carer	2
Provider	1
Total	23

Several themes emerged during the workshop discussions, around the criteria for determining which innovations should be prioritised for evaluation. There was a desire for a range of types of innovations to be evaluated, to include a mix of community-centred, individual-centred and technological innovations. Participants wanted to ensure that some innovations that focused on community-centred support and connecting communities were prioritised. They also wanted innovations that support individuals and families to maintain independence, e.g. innovations that focus on prevention, self-directed support or helping people to do more for themselves. Technological innovations such as apps and web-based interventions were also considered necessary to be included, although some participants contested the suitability of technological innovations for certain user groups and their suitability for rapid evaluation. To facilitate the desire for a mix, participants decided to prioritise innovations that they considered represented groups of similar innovations. For example, among all the technological innovations, one group prioritised the one that was perceived to be the most sophisticated or potentially beneficial. Innovations that were seen to be breaking wholly new ground, were novel or shaking up the current social care system, were ranked higher.

There was discussion of the relative priority of evaluating innovations expected to be quality improving but also cost increasing, versus those focused on cost saving. One group queried whether we should be evaluating innovations that local authorities were unlikely to be able to afford to implement. But other participants considered it preferable to prioritise evaluating innovations that might strengthen how social care services are delivered, ahead of innovations aimed at cost saving.

Innovations that appeared potentially generalisable but were currently lacking supporting evidence were prioritised over those that were known to have been evaluated or currently being evaluated. However, there were varying views on what counted as having enough evidence: e.g. the extent to which innovations had been evaluated for use with different target groups within a given population or evaluated in other countries (whereby findings might not be wholly applicable to UK settings). Some participants accorded higher priority to innovations that focused on underrepresented or relatively neglected groups with unmet needs (such as individuals living with brain injury, or prisoners).

The top five innovations to prioritise for evaluation that were identified at the end of the workshop are described in Walton et al. (2019) [23]. These innovations (listed here in alphabetical order) were: (i) Care coordination for dementia in the community, (ii) Family group conferencing, (iii) Greenwich prisons social care, (iv) Local area coordination and (v) MySense.AI. Taken together the top five span a wide diversity of innovation types. All aim mainly to improve care quality rather than save costs. They include two innovations around ways of coordinating care locally, including community assets; one focused at the individual level, looking at how to improve planning of individualised care; one was a technology-focused innovation; and one aimed at a particularly under-served group of the population, namely prisoners.

Fifteen out of 23 (65%) participants provided feedback. Feedback indicated that participants welcomed the information sent prior to the workshops about the innovations and the plans for the workshop itself, although some participants felt there was less information on some innovations than on others and would have welcomed greater information (if available) as well as more time than one week to gather their thoughts prior to attending the workshop.

The key tensions expressed by participants were: (1) the application of the JLA format and reaching consensus in a single workshop; and (2) achieving a balance of voices in discussions to prioritise innovations. Some participants felt there was some ‘disconnect’ between the small group discussions in the first two stages of prioritisation at the workshop, and the final, plenary discussion. The lack of pre-set criteria left some participants feeling uneasy. Some participants considered that some of the discussions had, despite the facilitators’ efforts to ensure inclusion, been disproportionately driven by “stronger characters” who were more informed and knowledgeable about particular innovations. Hence, there were notable differences between how innovations were prioritised in a small group and the final order agreed in the large group discussion. Nevertheless, participants welcomed the opportunity to contribute in a workshop attended by a range of individuals committed to improving services in adult social care; and, while many have critiqued the methodology of an adapted JLA process, participants overall felt the workshop achieved a sufficient level of inclusivity and consensus, and was able to prioritise innovations based on informative discussions.

## Discussion and conclusions

An established, dialogue-based model for inclusive research priority setting, the JLA approach has been used internationally in over 100 areas of health and social care [24]. JLA Priority Setting Partnerships (PSP) focus on the prioritisation of health research questions, facilitating a process that involves multiple stakeholders in a 12- to 18-month process. In order to rapidly to set priorities for the evaluation of innovations in social care and social work, the JLA framework [18, 20] was adapted with the aim of being done rapidly, but still with input from a range of stakeholder groups, including people who use services, carers, practitioners, providers, commissioners and researchers. In this section, we review the adaptations we made to the JLA process and the steps we took to try and uphold the rigour of the exercise. We examine the implications of undertaking rapid prioritisation, including the impact on the engagement of stakeholders and on the innovations prioritised for rapid evaluation.

The rapid priority setting exercise was delivered in four months’ elapsed time, from commencing initial planning to reporting the priorities to NIHR. This rapid exercise took a lot less time than most JLA priority setting processes which usually take 12–18 months (from design through to comprehensive checking processes) [25–27].Project management was a key factor in achieving speed. A multi-disciplinary project team was established to design and run the project, including coordination and administration support, evaluation and research expertise, social care and social work research expertise, and priority setting methods experience. JLA PSPs are similarly operationally supported by a project team but, unlike our rapid adaptation, they are also overseen and led by a steering group involving service users and practitioners [25–27]. The team for the rapid prioritisation was able to focus on the innovation identification and priority setting and convene meetings quickly and regularly, including overseeing the shortlisting. Additional professional expertise was brought in on an ad hoc basis, including calling on colleagues to help identify networks for recruiting workshop participants, and to sense-check the selection of innovations shortlisted for discussion at the workshop.

One reason for adapting the JLA method, as opposed to any other priority setting method, was to draw on its methods for involving diverse stakeholders in the decision-making process. It was agreed that the results should be shaped by a range of groups, including people with lived experience of accessing social care and social work services, and their carers. However, undertaking a rapid prioritisation did affect the ability to engage with service users in the earlier stages of the process (as is recommended in JLA PSPs) (e.g. [25–27]). For example, no individual service users submitted innovations to the consultation seeking candidate innovations. The short time available meant that there was no opportunity to develop a separate consultation approach tailored for this group (which was something the JLA Adult Social Work PSP did) or to develop relationships with individuals and groups who could provide access to those who use services and their carers. Instead, the consultation relied on the project team’s existing networks, with no opportunity to build relationships with community groups and secure buy-in and participation that way. Individuals with lived experience were recruited to the priority setting workshop and actively influenced the outcomes of that, but these individuals tended to have established links to advocacy groups and other involvement activities. More vulnerable potential service users, including people with learning disabilities and cognitive impairment, were not involved. Participants were mindful of this and tried to represent the interests of the under-represented where possible.

Achieving buy-in was potentially a challenge. This is consistent with previous research which has highlighted the challenges of engagement [28]. JLA PSPs that focus on single healthcare conditions can engage with established patient and clinician communities who have common experiences, knowledge, terminology and a clear understanding of their clinical area (e.g. [29]). Their vested interest in the topic helps secure their buy-in. A broad topic such as adult social care and social work does not involve a single constituent group. Undertaking rapid priority setting on this topic meant that there was no time to build a partnership of engaged parties and participants, to develop shared understanding and establish common goals. Achieving engagement for social care may also be more challenging due to social care being a large and diverse topic. Securing engagement may be easier for rapid priority setting on narrower topics.

The rapidity of prioritisation may affect its results. In a full JLA priority setting exercise, the consultation to collect people’s ‘unanswered questions’ lasts at least three months (e.g. [25–27]). A further two to three months are then spent collating that data, creating summary questions and checking them against systematic reviews and guidelines, in order to remove questions that do not require further research. This is supported by a patient- and-clinician-led Steering Group, which reviews the interpretation of the raw data and the development and wording of the summary research questions, before those questions go back into the public domain for prioritisation. In the exercise presented in this paper, the consultation asked for people to suggest known, named innovations in social care or social work that could benefit from evaluation. This approach required people to understand the notion of innovation and to have enough knowledge to be able to identify one or more. Taking a rapid approach meant that the search for innovations could not be comprehensive, as not all current innovations would be known to the individuals consulted.

There was not time prior to the workshop to check more than cursorily the extent to which the shortlisted innovations had been evaluated already, although this was done more thoroughly subsequently for the top five proposed priorities for evaluation. While colleagues in the field were asked to sense-check the list, it is possible that some items went forward to the prioritisation workshop that were low priorities for further evaluation. Indeed, during the priority setting workshop, one innovation that had been a high priority in some of the initial group discussions, was deprioritised in the final, plenary, workshop session when a participant had the opportunity to inform all participants of an ongoing evaluation. Had there been more time before the workshop, this could have been avoided: the item would not have made it to the workshop. Future rapid prioritisation exercises could develop a plan for managing this, at the cost of more elapsed time and more researcher inputs.

Whilst criteria were used by the research team to reduce the very long initial list to a list of 20 for consideration in the workshop (see Additional file 1: S2), we did not wish to constrain workshop participants with pre-set criteria. Some workshop participants were concerned about the (deliberate, in the JLA method) absence of pre-set criteria for determining priorities for evaluation. The aim was to permit the participants to generate the criteria according to their different perspectives. This aim appears to have been achieved and no participants, despite some expressing concerns in feedback, proved unable or unwilling to contribute actively to the prioritisation discussions. It was also noted by some participants in their post-workshop feedback, that some voices had proved more powerful than others in discussions, particularly at the final, plenary, stage of the workshop. There was no suggestion of disrespectful behaviour by any participants, and it is to be expected that those with more knowledge of an item are more likely to speak, and to speak more, about it. Nevertheless, this is an important and sensitive issue that requires careful and deliberate mitigation by workshop facilitators.

One limitation of this process was that the speed with which we identified innovations meant that service users were unable to suggest innovations at the beginning of the process and that the responses to our call for innovations may have been limited, or potentially biased. Therefore, some innovations may have been missed. Given the nature of our rapid prioritisation process (which included a call for innovations), it is likely that service user involvement at the beginning of the process may have required a longer process including discussion groups. However, we tried to ensure that we identified as many innovations as possible and we conducted checks to ensure that the risk of bias or missing innovations were minimised (e.g. consulting experts). A further limitation is that we were unable to include input from service users on the analysis and reporting of the prioritisation process.

Despite the limitations discussed here, we managed to ensure that innovations were suggested, in rapid responses, by a wide range of organisations and individuals with varying roles within these organisations and the adult social care and social work field. We were then able to adapt and apply an established, robust method for priority setting—that used by the JLA [18]—rapidly and with clear outcomes agreed by a multi-stakeholder group.

We have outlined a systematic but pragmatic method that other researchers who would like to undertake rapid prioritisation processes could imitate. The findings from the specific prioritisation of social care and social work innovations for evaluation can be used to inform the selection of social care innovations to be evaluated. This will support increasingly effective social care and social work to the benefit of service users in future.

We conclude that a rapid version of a priority setting method such as the JLA’s may be helpful when engaging in rapid prioritisation processes. Our experience indicates that an adapted version of this prioritisation method was feasible for identifying priorities in a rapid and systematic way, with limitations. However, users of this approach should be aware of the implications and the compromises it entails.

## Supplementary Information

**Additional file 1.** **Supplementary file 1.** Flow chart outlining the four steps involved in our approach. **Supplementary file 2.** Criteria for inclusion in reduced list.

## Data Availability

The datasets used and/or analysed during the current study are available from the corresponding author on reasonable request.

## Abbreviations

JLA: James Lind Alliance
NHS: National Health Service
NIHR: National Institute for Health Research
PSP: Priority Setting Partnership
REPRISE: Reporting guideline for PRIority Setting
SCIE: Social Care Institute for Excellence
TLAP: Think Local Act Personal
WHO: World Health Organisation
