# Supplementary material for: Rapid prioritisation of topics for rapid evaluation: the case of innovations in adult social care and social work
Source: Health Res Policy Syst. 2021 Mar 10;19:34. doi: 10.1186/s12961-021-00693-2 (PMC7944624; doi:10.1186/s12961-021-00693-2)
Supplement: Supplementary file 1 — Additional file 1. Supplementary file 1. Flow chart outlining the four steps involved in our approach. Supplementary file 2. Criteria for inclusion in reduced list. [file 12961_2021_693_MOESM1_ESM.docx]

**Supplementary materials**

*Supplementary file 1.* Flow chart outlining the four steps involved in our approach


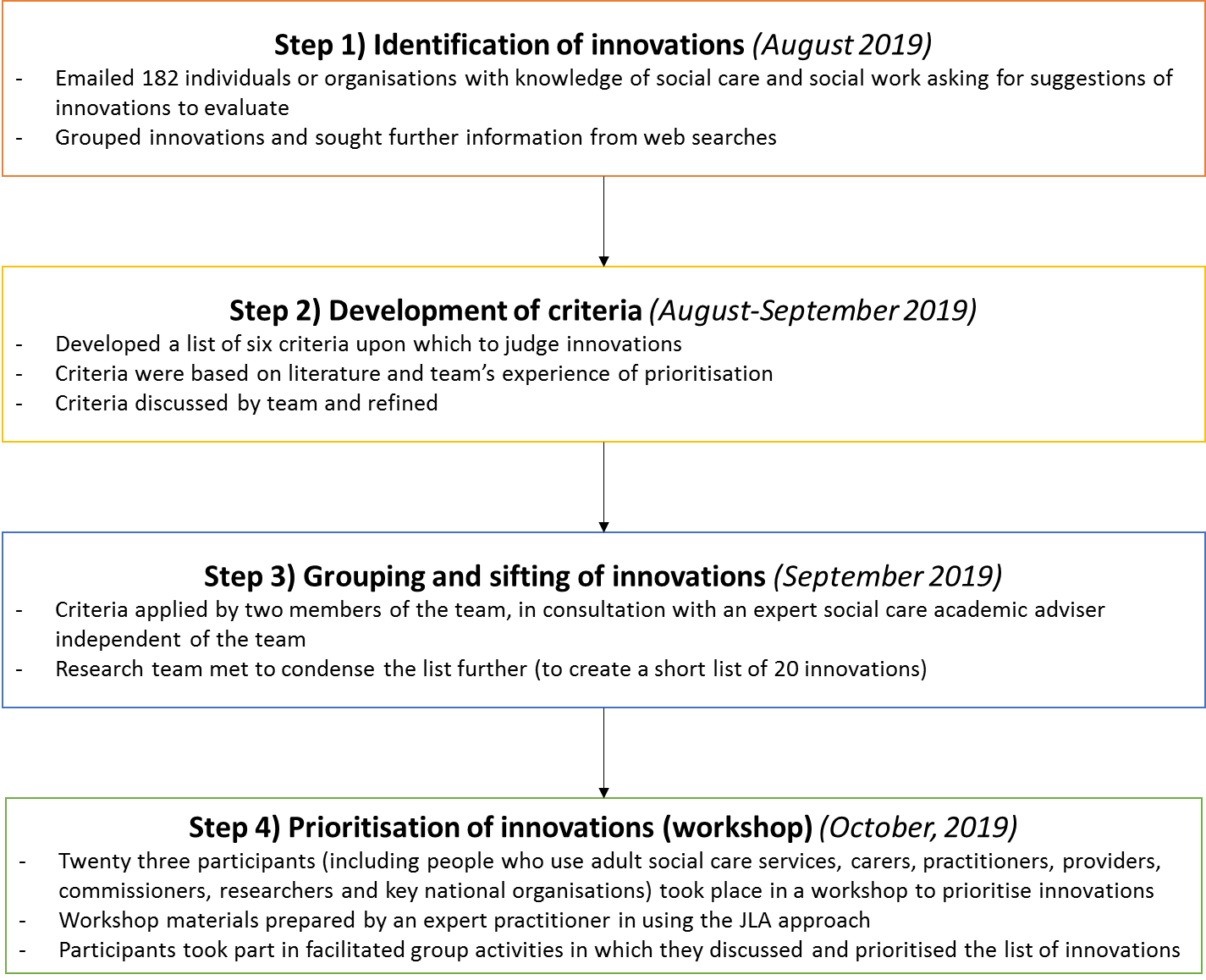


*Supplementary file 2.* Criteria for inclusion in reduced list

| **Criterion** | **Description** |
| --- | --- |
| **1. Adult social care and social work only** | Innovation must target adult social care and social work alone or in combination with health care and can be in any setting. Innovations targeting only children or young people should be excluded. Adult social care is personal and practical care and support that people may need because of their age, illness, cognition, disability or other circumstances. It includes support for family members or other unpaid carers. |
| **2. Focuses on a relevant outcome** | The innovation must focus on one or more relevant outcomes for social care, such as:   - To help people remain independent - To retain their dignity - To achieve a better quality of life - To safeguard vulnerable individuals from abuse and neglect - To deliver social care more efficiently |
| **3. Taking place somewhere in the UK** | To be evaluable, the innovation must already be taking place somewhere. The UK focus was in line with the remit for the study from the NIHR. |
| **4. Respondent provided enough detail to understand what the innovation is** | To enable prioritisation for evaluation, some information of the following kinds is needed:   - What the innovation entails - Existing evidence-base for it - Desired outcomes - Scale (e.g. one care home or one care home group or much broader?) - Geographical reach - When the innovation emerged - Potential magnitude of benefit |
| **5. Amenable to evaluation** | - Are there existing evaluations (underway or completed)? - If yes, are there remaining evidence gaps that an evaluation might fill? - Is there potential for broader/national learning, beyond the site(s) involved - Is there a need in social care for learning about this innovation? |
| **6. Amenable to *rapid* evaluation** | - Could the innovation be more appropriately evaluated in a longer time frame through commissioned research? - Is the innovation in operation/being trialled currently? (I.e. is there is something concrete to evaluate?) - Is local buy-in for an evaluation likely? - Is the innovation well defined? - Is it a good time to evaluate (e.g. are there benefits or risks to early evaluation)? |
